# Oral Nutritional Supplementation Improves Growth in Children at Malnutrition Risk and with Picky Eating Behaviors

**DOI:** 10.3390/nu13103590

**Published:** 2021-10-14

**Authors:** Deepti Khanna, Menaka Yalawar, Pinupa Venkata Saibaba, Shirish Bhatnagar, Apurba Ghosh, Pramod Jog, Anuradha Vaman Khadilkar, Bala Kishore, Anil Kumar Paruchuri, Prahalad D. Pote, Ravi D. Mandyam, Sandeep Shinde, Atish Shah, Dieu T. T. Huynh

**Affiliations:** 1 Abbott Nutrition, Research & Development India, 15th Floor, Godrej BKC Plot–C, “G” Block, Bandra Kurla Complex, Bandra East, Mumbai 400051, Maharashtra, India; 2 Statistical Services, Cognizant Technology Solutions India Private Limited, Manyata Business Park, Nagavara, Bengaluru 560045, Karnataka, India; menaka.shekarappa@abbott.com (M.Y.); venkatasaibaba.pinupa@abbott.com (P.V.S.); 3 Ajanta Research Centre, Ajanta Hospital & IVF Centre, 765, ABC Complex, Kanpur Road, Alambagh, Lucknow 226005, Uttar Pradesh, India; drshrishbhatnagar@gmail.com; 4 Institute of Child Health, Ground Floor, 11, Biresh Guha Street, Kolkata 700017, West Bengal, India; apurbaghosh@yahoo.com; 5 Medipoint Hospital, S. No. 241/1, New D.P. Road, Aundh, Pune 411007, Maharashtra, India; dr_pramodjog@yahoo.co.in; 6 Jehangir Clinical Development Centre, Jehangir Hospital, 32, Sassoon Road, Near Pune Station, Pune 411001, Maharashtra, India; anuradhavkhadilkar@gmail.com; 7 Saint Theresa’s Hospital, Erragadda, Sanath Nagar, Hyderabad 500018, Telangana, India; baki2004@gmail.com; 8 Praveen Cardiac Centre, Moghalrajpuram Madhu Garden bus stop, No. 5 Bus Route, Vijayawada 520010, Andhra Pradesh, India; paruchuriak70@gmail.com; 9 Noble Hospital Private Limited, 153, Magarpatta City Road, Hadapsar, Pune 411013, Maharashtra, India; drppote@gmail.com; 10 JSS Academy of Higher Education and Research, Mysuru 570004, Karnataka, India; ravimdped@gmail.com; 11 Pune Sterling Multispecialty Hospital, Sector 27, Near Bhel Chowk, Pradhikiran, Nigdi, Pune 411044, Maharashtra, India; drsandeepshinde@gmail.com; 12 Sangini Hospital, Sangini Complex, Near Parimal Crossing, Ahmedabad 380006, Gujarat, India; dr_atish78@yahoo.com; 13 Abbott Nutrition Research and Development Asia-Pacific Center, Singapore 138668, Singapore; dieu.huynh@abbott.com

**Keywords:** children, growth, picky eating, malnutrition, oral nutritional supplements

## Abstract

The problem of poor nutrition with impaired growth persists in young children worldwide, including in India, where wasting occurs in 20% of urban children (<5 years). Exacerbating this problem, some children are described by their parent as a picky eater with behaviors such as eating limited food and unwillingness to try new foods. Timely intervention can help prevent nutritional decline and promote growth recovery; oral nutritional supplements (ONS) and dietary counseling (DC) are commonly used. The present study aimed to determine the effects of ONS along with DC on growth in comparison with the effects of DC only. Enrolled children (N = 321) were >24 to ≤48 months old, at malnutrition risk (weight-for-height percentile 3rd to 15th), and described as a picky eater by their parent. Enrollees were randomized to one of the three groups (N = 107 per group): ONS1 + DC; ONS2 + DC; and DC only. From day 1 to day 90, study findings showed significant increases in weight-for-height percentile for ONS1 + DC and for ONS2 + DC interventions, as compared to DC only (*p* = 0.0086 for both). There was no significant difference between the two ONS groups. Anthropometric measurements (weight and body mass index) also increased significantly over time for the two ONS groups (versus DC only, *p* < 0.05), while ONS1 + DC significantly improved mid-upper-arm circumference (*p* < 0.05 versus DC only), as well. ONS groups showed a trend toward greater height gain when compared to DC only group, but the differences were not significant within the study interval. For young Indian children with nutritional risk and picky eating behaviors, our findings showed that a 90-day nutritional intervention with either ONS1 or ONS2, along with DC, promoted catch-up growth more effectively than did DC alone.

## 1. Introduction

In children, growth is a recognized indicator of nutritional status [1]. The number of children worldwide with poor growth remains alarmingly high today [2,3,4]. Global growth statistics recently showed nearly 50 million children under 5 years old were wasted (low weight-for-height), and 149 million were stunted (low height-for-age)—with Asia and Africa bearing the greatest share [4]. Although such growth markers are improving in India, about 20% of young children (<5 years) were recently reported as wasted and 36% as underweight [5,6,7]. If undernutrition and growth faltering are not addressed in early childhood, some consequences are irreversible and can negatively affect the ability of these children to learn during their school years and to reach their full potential as productive adults [4,8,9].

To facilitate early detection of problems, the World Health Organization has developed growth standards for children by age and sex [1]. Three often-used anthropometric indices are weight-for-age, height-for-age, and weight-for-height, which are expressed in Z-scores (standard deviation values) or as percentiles [1,10]. Other measures—body mass index (BMI)-for-age and arm circumference-for-age-are also used to evaluate growth [1]. A Z-score between −2 and −1 (corresponding approximately to the 3rd to 15th percentile) represents mildly poor growth and corresponding mild undernutrition (also called malnutrition risk), while a Z-score between −3 and −2 indicates moderate undernutrition, and <−3 signifies severe undernutrition [11]. Likewise, wasting is defined by weight-for-height Z-scores (WHZ), stunting by height-for-age Z-scores (HAZ), and underweight by weight-for-age Z-scores (WAZ).

Children may not get sufficient healthful, nutritious foods for normal growth for a variety of reasons—lack of information or education on healthy food supplies, limited access to healthy foods, or cultural preferences of the family [12]. As well, picky eating behaviors put some young children at real nutritional risk [13,14,15,16]. Children described as picky eaters were more likely than non-picky eaters to be underweight [15,17]. Nutritional shortfalls not only increase a child’s risk for compromised growth but also increase vulnerability to infectious pathogens, in turn heightening risk for illnesses [18]. In developing countries, children who were just mildly malnourished (weight-for-height Z scores between −2 and −1) [19] were at greater risk of morbidity and mortality [20].

Around the world, and especially in developing countries, health professionals and parents alike are concerned about poor health outcomes related to undernutrition (wasting, stunting, and underweight) in early childhood [19,21]. Importantly, nutritional intervention for young children with malnutrition risk can help prevent further growth faltering and promote catch-up growth [22]. When a child with evidence of wasting also displays picky eating habits, there is a potential need for nutritional intervention to support adequate growth and to improve health outcomes. This report describes our study in India of young children who were mildly undernourished (defined as weight-for-height Z-score between −2 and −1, i.e., between the 3rd and 15th percentiles) and picky eaters but were otherwise healthy. In this three-arm study, we evaluated the impact of a 90-day intervention with one of two oral nutritional supplement formulations (ONS1 and ONS2) plus dietary counseling (DC) on growth, as compared with the impact of a 90-day intervention of DC only.

## 2. Materials and Methods

The study protocol, all amendments, and the informed consent form were reviewed and approved by the Independent Ethics Committee/Institutional Review Boards of all 10 study hospitals. Parents or guardians of subjects voluntarily gave written informed consent prior to enrollment. The study was performed in accordance with the protocol, Good Clinical Practice (GCP) guidelines (Vijayananthan and Nawawi 2008), local regulations governing clinical study conduct, and the ethical principles that have their origin in the Declaration of Helsinki. The study was registered at clinicaltrials.gov in the US (Abbott Nutrition 2015) (NCT02523027) and with Clinical Trials Registry India (CTRI/2015/10/006330 (Abbott Nutrition International India 2015).

This multi-center (10 hospitals and clinics in urban or semi-urban India), prospective, randomized, double-blinded study used two parallel treatment groups and one open-labeled control group. The groups were: (i) ONS1 + DC, (ii) ONS2 + DC, and (iii) control DC only. A parent/guardian gave each child the designated ONS and attended DC sessions on feeding his or her child. The study collected data at baseline and four follow-up time points in the 90-day intervention period, i.e., Days 1 (baseline), 7, 30, 60, and 90. At Day 1, the parents/guardians in experimental groups received their respective ONS1 + DC or ONS2 + DC, whereas parents/guardians of children in the control group received only DC. After Day 1, experimental group children were given their respective ONS daily for 90 days. Parents/guardians of children in all groups underwent additional DC sessions at Days 7, 30, and 60.

ONS1 and ONS2 (PediaSure and PediaSure Advance, respectively, Abbott Healthcare Private Limited, Mumbai, India) had similar nutrient compositions, but ONS1 was milk-based, while ONS2 was lactose-free, as detailed in Table S1 (Supplementary Materials). Both formulations contained 3 macronutrients with matching levels of protein (at 12% of energy), 28 vitamins and minerals, and the pre-biotic fiber fructo-oligosaccharide (FOS). The fat energy percent was higher and carbohydrate energy percent was lower in ONS 2 as compared to ONS 1.

For inclusion, study subjects were of either sex, >24 months but ≤48 months of age at day 1, and each had a weight-for-height between the 3rd and 15th percentiles according to the WHO Growth Standards 2006 (WHO 2006, Mehta, Corkins et al. 2013). Included subjects were designated by a parent or guardian as a picky eater when the child met at least two of the following criteria: (a) eats only a limited number of foods, (b) is unwilling to try new foods, (c) refuses to eat vegetables or foods from other food groups, (d) shows strong food likes and dislikes, and (e) has behaviors that disrupt mealtime. Additionally, a child was included only if he or she habitually drank at least 1 glass milk (approx. 200 mL) per day. Each child’s parent or guardian agreed to abstain from giving non-study nutritional supplements (vitamin and mineral supplements, micro-nutrient-fortified beverages, and ONS other than the study products) during the intervention phase.

Subjects were excluded from the study if they had been diagnosed or were known to be: (a) be lactose intolerant and galactosemic; (b) be allergic or intolerant to any ingredient found in the study product; (c) have a current acute or chronic infection including but not restricted to respiratory infection, diarrhea, Hepatitis B or C, HIV infection or tuberculosis; (d) have severe gastrointestinal disorders including celiac disease, short bowel syndrome, pancreatic insufficiency, or cystic fibrosis; (e) have a diagnosis of neoplastic, renal, hepatic or cardiovascular disease, hormonal or metabolic disorders, congenital disease or genetic disorders such as atrial or ventricular wall defects, or Down’s syndrome, infantile anorexia nervosa, developmental disability, including physical disorders such as cerebral palsy, or developmental delay (f) diagnosed with disorders of hemoglobin structure, function or synthesis according to medical records or parent/guardian report, or (g) have had a clinically significant nutritional deficiency requiring specific treatment with another nutritional supplement (other than the study product) or (h) have any other clinically significant medical condition, which, in the investigator’s opinion, made the child unsuitable for inclusion in the study.

We assigned enrolled subjects and randomly allocated each to a treatment group. Randomization schedules were computer-generated using a pseudo-random permuted blocks algorithm. Randomization was stratified by study center. For blinding of treatments, we placed ONS1 and 2 product pouches in a white opaque jar labeled with a seven-character product code. Neither investigators, their staff, members of Abbott Nutrition’s staff, nor families of subjects were informed of the ONS identity during the study period. Study center personnel did not analyze the ingredients of the ONS formulations, nor did they seek to identify the ONS products. The frequency of follow-up and outcome measurements were identical in both treatment groups.

For each study subject, a measuring shaker (calibrated to 200 mL) was provided for reconstitution of ONS powder and for measuring ONS consumption. Compliance was calculated as ONS intake divided by recommended intake over the study period. Subjects in the ONS groups took a minimum of 1 serving/day or a maximum of 2/day for the 90-day intervention interval. Compliance was assessed via diary records kept by the parent/guardian; subjects were considered compliant if they consumed at least 75% of the recommended ONS intake.

The primary outcome measure was change in weight-for-height percentile from Day 1 to Day 90. Other outcomes were changes in anthropometric indices from Day 1 (baseline) to Days 30, 60, and 90. Anthropometric measurements of weight, height, body mass index (BMI), and mid-upper-arm circumference (MUAC) were made on Days 1, 30, 60, and 90 for all three groups; weight was also measured on Day 7. All measurements were performed by study staff who had been trained to use standardized methods. Weight was measured (in minimal clothes, with shoes and jackets removed) using calibrated electronic weighing scales (Phoenix PPS-160) and was recorded to the nearest 0.1 kg. Standing height was measured (shoes, hat removed) using a height stadiometer (Escala) and was recorded to the nearest 0.1 cm. BMI was calculated as BMI = weight (in kg) ÷ height (in m^2^). MUAC was determined with a non-stretchable measuring tape and was recorded to the nearest millimeter. We expressed these measures as sex–age-specific Z-scores and percentiles based on the WHO Child Growth Standards 2006 (WHO 2006, WHO 2007) for weight-for-height, weight-for-age, BMI-for-age, height-for-age, and MUAC-for-age.

We also assessed the average energy consumption based on the parents’/guardians’ 24 h dietary recall reports. A trained dietitian provided dietary counseling, which included guidelines for consuming a balanced diet and a wide variety of foods from various food groups, improving the quality of the diet, and for meeting the child’s daily nutritional requirements. From each child’s parent or guardian, the dietitian collected one 24 h dietary recall at baseline and at each follow-up time point by an in-person, on-site interview. For consistency, a trained dietitian at the Madras Diabetes Research Foundation (MDRF), Chennai, reviewed the energy consumption report from all study sites. Comparison with the Estimated Average energy Requirement (EAR) 2020 for 1–3-year-olds (1110 Kcal/d) and 4–6-year-olds (1360 Kcal/d) have been made, as subjects in this study were > 2 and ≤ 4 years.

Adverse events (AEs) were reported by parents and caregivers and verified by study physicians. All diagnoses of reported AEs were standardized using the Medical Dictionary of Regulatory Activities (MedRA) version 21.1.

All randomized subjects who consumed any amount of the study product were included in the intention-to-treat (ITT) cohort, and ITT analyses were made on an ITT basis. Continuous variables at baseline and post-intervention were analyzed using parametric tests (analysis of covariance, ANCOVA) unless the distribution of the variable was declared non-normal, in which case a non-parametric test (Wilcoxon rank sum test) was used. Residuals from the parametric analysis were used to check for deviation from normality by a combination of methods (stem-and-leaf plot, normality plot, and Shapiro–Wilk test). All hypothesis testing, except for the tests for interaction and normality, were done using 2-sided, 0.05 level tests of significance. The step-down Bonferroni (Holm) procedure was used to adjust the significance levels for multiple comparisons. For parametric analyses of post-intervention growth variables, the covariates age, study site, and gender were added, and interactions between study site and gender were included. For analyses of baseline variables with ANCOVA, age, gender, and study site were used as covariates. For growth variables, changes-from-baseline in each treatment group were also analyzed using a one-sample paired t-test or a signed rank test (if declared non-normal). Categorical variables were analyzed using Cochran–Mantel–Haenszel, Chi-square, or Fisher’s exact test. Tests for interactions and normality were done using 2-sided, 0.10- and 0.001-levels, respectively. When an interaction was significant, the step-down Bonferroni (Holm) procedure was used to adjust the significance levels for multiple comparisons. Statistical software SAS release 9.3 (SAS Institute Inc., Cary, North Carolina, USA) was used. A *p*-value < 0.05 was considered statistically significant.

## 3. Results

**Figure 1:**
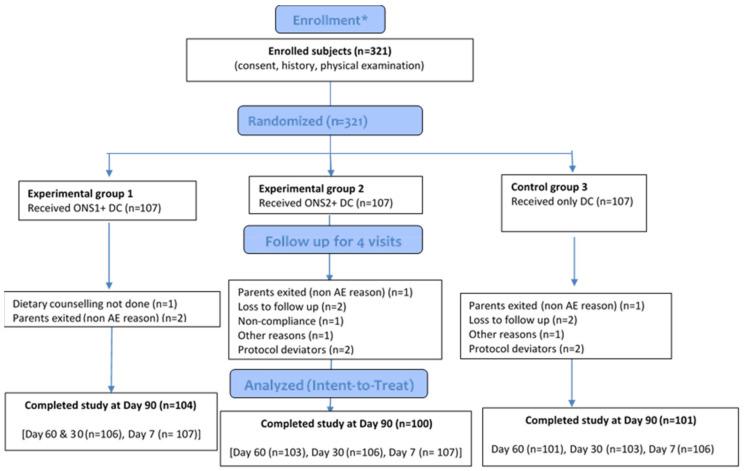
CONSORT 2010 flow diagram * Enrollment and randomization was at baseline/visit 1. Baseline and demographic characteristics were reported for all randomized subjects. (ONS: Oral Nutritional Supplement; DC: Dietary Counseling; AE: Adverse Event).

A total of 321 eligible children were included in the study; each child was randomly assigned to one of three study groups (N = 107 children per group) as shown in the CONSORT Flow Diagram (Figure 1) (Schulz, Altman et al. 2010). For study eligibility, each subject was between ages 24 and 48 months, was mildly wasted, and was described by a parent–guardian as a picky eater. At the time of recruitment, most subjects met the inclusion criteria. Four subjects were found to be ineligible, as their weight-for-height was not between the 3rd and 15th percentiles on Day 1. Of the ITT subjects, 21 subjects were not evaluable, mainly due to non-compliance with consuming the study product. Data were collected from June 2016 to February 2017; the intervention interval was 90 days for each included subject.

### 3.1. Baseline Socio-Demographic and Anthropometric Characteristics

For baseline socio-economic characteristics, there were no significant between-group differences (ONS1 + DC, ONS2 + DC, and DC only) for gender distribution, age at enrollment, family composition and structure, maternal/paternal mean age, maternal/paternal schooling years, and subjects’ habitual milk drinking. The full study enrolled a higher proportion of boys (60%) than girls (40%); the mean age at enrollment age was 2.93 years (Table 1). Two-adult families were predominant (57%) for all subjects, with the next largest proportion as three- or four-adult families (28%). Most families had one child ≤5 years in the family (63%), and most subjects were from nuclear families (71%) with 64% of mothers voluntarily not working and 98% of fathers employed full time. The mean age of mothers was 28 years, while that of fathers was 32.7 years. Baseline anthropometric characteristics in the study group (N = 321) showed mean values as weight of 11.11 kg, height of 88.78 cm, BMI of 14.06 kg/m^2^, and MUAC of 14.08 cm. At baseline, there were no significant differences between the three groups for weight, height, BMI, and MUAC after adjusting for the effects of age at enrollment, site, and gender (Table 1).

There were no significant between-group differences for weight-for-height, weight-for-age, height-for-age, and MUAC-for-age Z-scores and percentiles. However, there was a significant difference between the two ONS groups for the weight-for-height Z-score. Baseline anthropometric characteristics were thus comparable among the three study groups.

### 3.2. Primary Outcome: Change in Weight-for-Height Percentile

**Figure 2:**
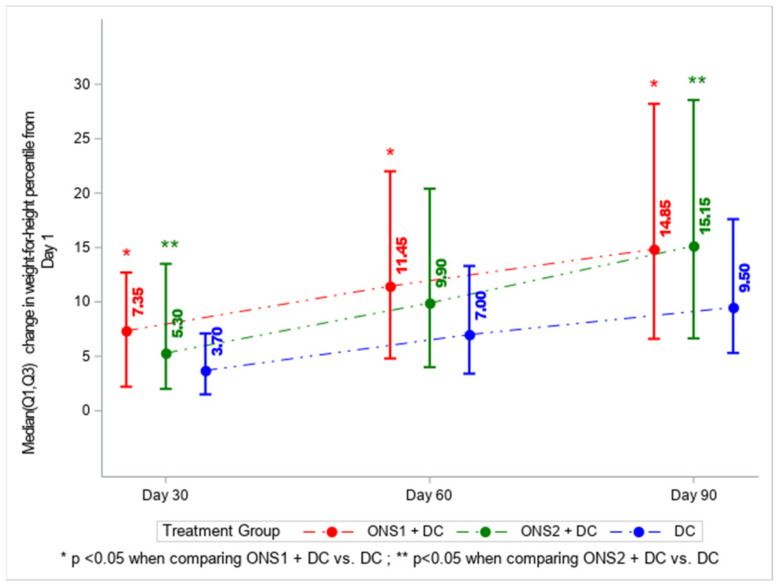
Change in weight-for-height percentile from Day 1 to Days 30, 60, 90 by intervention group: ONS1 + DC, ONS2 + DC, or DC only. *p*-values were calculated using the Wilcoxon rank sum test.

Change in weight-for-height percentile from Day 1 to Day 90 showed significant increases for the ONS1 + DC group and for the ONS2 + DC group, as compared to the group with DC only (*p* = 0.0086 for both). There was no significant difference between ONS1 and ONS2 treatment groups (*p* = 0.935) at 90 days (Figure 2).

### 3.3. Absolute Change in Weight, Height, BMI, and MUAC across Time Points

**Figure 3:**
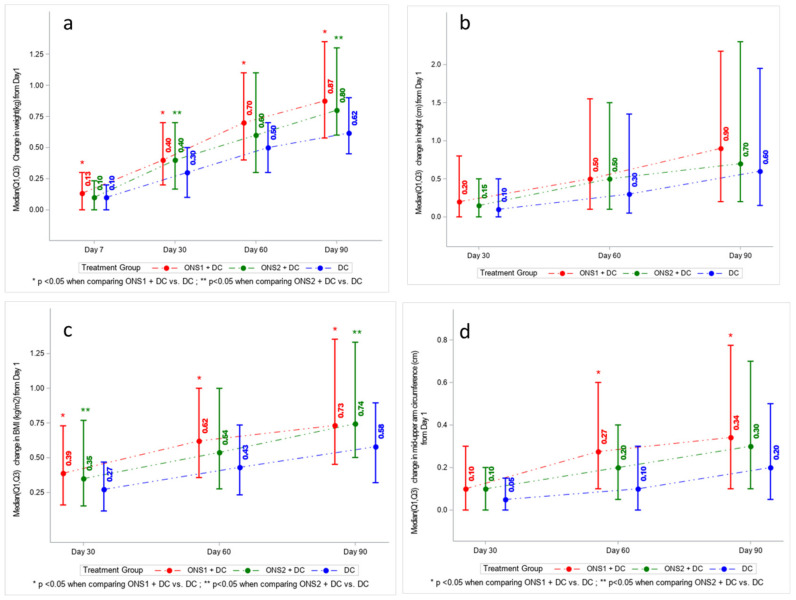
Change from Day 1 to Days 30, 60, 90 by study group for (**a**) weight, (**b**) height, (**c**) Body Mass Index, and (**d**) Mid Upper Arm Circumference.

In terms of anthropometric outcomes, we determined changes in weight, height, BMI, and MUAC over a 90-day interval with measurements made on days 1, 7, 30, 60, and 90 (Figure 3a–d). For changes in weight (Figure 3a), significant differences were observed between ONS1 + DC versus DC only group from Day 1 to Day 7 (*p* = 0.0222), Day 30 (*p* = 0.0015), Day 60 (*p* = 0.0043), and Day 90 (*p* = 0.0012). Significant differences were observed between the ONS2 + DC group versus DC only group from Day 1 to Day 30 (*p* = 0.0140) and Day 90 (*p* = 0.0012). For changes in height (Figure 3b), between-group differences were not statistically significant over the 90-day time course. However, a trend for both ONS1 + DC and ONS2 + DC showing a higher median change in height (0.9 cm, *p* = 0.52 and 0.7 cm, *p* = 0.79, respectively) compared to DC only (0.6 cm) at 90 days was observed. For BMI (Figure 3c), significant differences in changes were observed between ONS1 + DC versus DC only from Day 1 to Day 30 (*p* = 0.0172), Day 60 (*p* = 0.0075) and Day 90 (*p* = 0.0185). Significant BMI differences were observed between ONS2 + DC versus DC only from Day 1 to Day 30 (*p* = 0.0358) and Day 90 (*p* = 0.0064). For MUAC, significant differences in changes were observed from Day 1 to Day 60 (*p* = 0.0230) and Day 90 (*p* = 0.0418) between ONS1 + DC versus DC only (Figure 3d). For all the anthropometric measurements of weight, height, BMI, and MUAC, there were no significant differences between the two ONS groups. For the full dataset, see Table S2.

### 3.4. Changes in Growth Indicators (as Percentiles and Z-Scores) across Time Points

**Figure 4:**
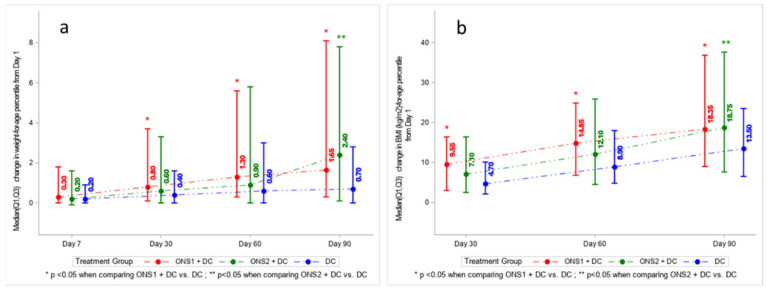
Changes in growth indicators expressed as percentiles: (**a**) Weight-for-age, (**b**) BMI-for-age.

As additional indicators of catch-up growth, we evaluated weight-for-age, BMI-for-age, weight-for-height, height-for-age, and MUAC-for-age percentiles by treatment group (full dataset in Table S3). Expressed as weight-for-age, we observed significant positive changes in percentile values for ONS1 + DC at 30, 60, and 90 days in comparison with the DC only group. For ONS2 + DC, changes in weight-for-age percentiles were significant only at Day 90 (Figure 4a). As BMI-for-age percentiles (Figure 4b), we observed significant positive differences in changes for ONS1 + DC at Days 30, 60, and 90, as compared to DC only. For ONS2 + DC, changes were significantly higher at Day 90. While the absolute height increased over time, we noted that the height-for-age percentile did not show an increase over time. For MUAC-for-age percentiles also, ONS1 + DC reported significant change from Day 1 to Day 90 (*p* = 0.0241) and Day 1 to Day 60 (*p* = 0.0342) (graphs not shown).

The weight-for-age, weight-for-height, height-for-age, BMI-for-age Z-scores and MUAC-for-age Z-scores were also assessed (Table S4). They showed similar patterns to percentile changes for both treatment groups, as compared to the DC only group.

### 3.5. Dietary Parameters

**Figure 5:**
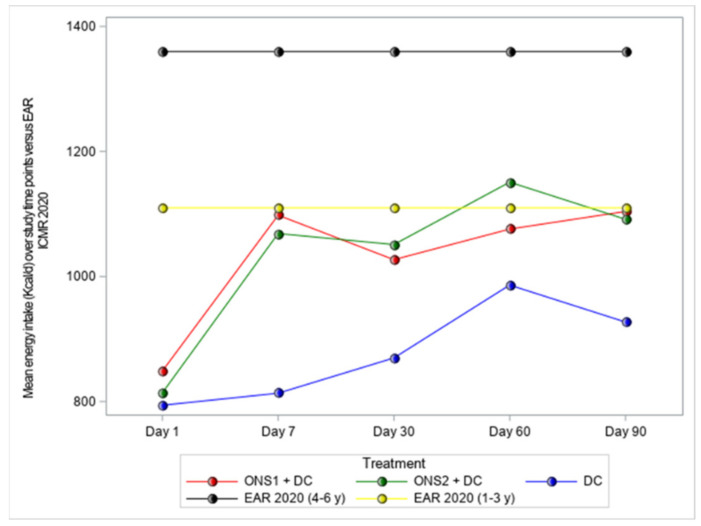
Mean energy intake (Kcal/d) compared to EAR 2020* at baseline and follow-up timepoints. * EAR for energy is equivalent to the estimated energy requirement (EER).

Energy intake was assessed based on the parents’/guardians’ 24 h dietary recall reported in interviews with the dietitian at follow-up timepoints. At baseline, energy intake for all groups was lower as compared to the Estimated Average energy Requirements (EAR 2020) [23], with no significant differences between the three groups. We found significant improvement in the average intakes of energy for both ONS1 + DC and ONS2 + DC vs. the control group for all the follow-up timepoints (Figure 5).

Compliance was assessed via diary records kept by the parent/guardian; subjects were considered compliant if they consumed at least 75% of the recommended ONS intake. There was high product compliance (approximately 99%) reported for the two experimental groups.

### 3.6. Adverse Events

Adverse events (AEs) were reported in 26 (24.3%), 33 (30.8%), and 20 (18.7%) children in the ONS1 + DC, the ONS2 + DC and the control groups, respectively. The most frequently reported AEs were respiratory and gastrointestinal tract events. There were no statistically significant differences in the number of AEs related to gastrointestinal or upper respiratory tract between the two ONS groups and between each ONS group and the control group (*p* > 0.05). Overall, there were no safety concerns associated with the consumption of the two products.

## 4. Discussion

Children at risk of malnutrition (Z-scores between −2 and −1, corresponding to 3rd to 15th anthropometric percentiles) are at increased risk of growth impairment, morbidities, and mortality; when such children also have picky eating behaviors, they are at even greater risk for poor growth and health outcomes. Timely and effective nutritional interventions are key to preventing further growth faltering and to promoting catch-up growth; ONS can be used to support adequate growth while promoting healthy eating behaviors (Box 1). In our current study on catch-up growth for such children, we examined the impact of two ONS formulas—one milk-based (ONS1) and the other lactose-free (ONS2)—over a 90-day interval. Both ONS formulas contained three macronutrients (with protein as 12% of energy), 28 vitamins and minerals, and the pre-biotic fiber fructo-oligosaccharide.

Findings in our study demonstrated the efficacy of parent- or guardian-guided daily ONS consumption by 2–4-year-old children who were at risk for malnutrition and had picky eating behaviors. With a 90-day ONS intervention, we found significant increases in WHO weight-for-height percentile (the primary outcome measure) from Day 1 to Day 90 for ONS1 + DC vs. DC alone, as well as for ONS2 + DC vs. DC only. We also found significant positive changes in other anthropometric indicators of growth when comparing ONS1 or ONS2 + DC versus DC only—weight and BMI as absolute measures, as well as weight-for-age and BMI-for-age as growth percentiles and as Z-scores. Low weight-for-height percentile indicates an acute malnutrition risk. In a review including 10 prospective studies with more than 53,000 children under 5 years old, mild wasting has been shown to be associated with about 62% greater risk of mortality due to infections when compared to normal children [20]. Hence, promoting catch-up growth in weight in mildly wasted children helps improve health outcomes.

We observed a trend for improvement in height over the 90-day study interval, but changes did not reach statistical significance. While wasting is a measure of an acute malnutrition, stunting is an indicator of chronic malnutrition, which is a result of a slow and cumulative process caused by sustained nutrient inadequacies [24]. Thus, nutritional intervention to promote catch-up growth in height takes longer as compared to catch-up weight. It should be noted that although children were enrolled based on mild wasting, they also had mild stunting. Most studies of nutritional repletion and height gain in stunted children used a time course of 6 months or longer [25].

For experimental groups, we observed high compliance with product intake (99%), which was associated with significant improvement in the average energy intakes. Taken together, our findings confirm and extend prior studies in which ONS, along with dietary counseling, was more effective than dietary counseling alone to promote catch-up growth.

Box 1 **Summary: study rationale, key study results, and take-home messages.**

- Children at risk of malnutrition (Z-score between −2 and −1) are at increased risk of impaired growth and other morbidities; when these children also have picky eating behaviors, they are at even greater risk for poor growth and health outcomes. For such children, it is important for healthcare providers to offer timely and effective interventions aimed at stopping further growth faltering and promoting catch-up growth.
- Daily ONS consumption, along with dietary counseling (DC) on healthier eating behaviors, can be used to help ensure adequate growth and to lessen risk for morbidities. In this study of catch-up growth, we examined the impact of two ONS formulas—one milk-based (ONS-1) and the other lactose-free (ONS-2)—used over a 90-day interval along with DC.
- Findings showed significant increases in weight-for-height percentile for ONS1 + DC and for ONS2 + DC interventions, as compared to DC only. Anthropometric measurements (weight and body mass index) also increased significantly over time for the two ONS groups.
- Growth parameters were comparable for both groups receiving ONS, suggesting the effects of these ONS formulas are similarly beneficial in promoting growth.
- ONS groups trended toward greater height gain but not full catch-up to WHO growth standards. Previous studies have shown that height improvement was only observed with intervention of longer duration (>6 months
- Findings in this study are consistent with previous studies in which ONS, along with dietary counseling, were more effective than dietary counseling alone in promoting catch-up growth in at-risk children.

### 4.1. Other Studies on Picky Eating Behaviors and Growth/Health Impairment in Young Children

It is not uncommon for parents around the world to describe their child as a picky eater—reporting behaviors such as limited variety of food the child is willing to eat, resisting new foods, avoiding certain foods, or having strong food preferences [14,26,27,28]. Longitudinal studies find that between 13% and 30% of children are considered picky at any given age, but fewer than half of those remain picky over a course of two years [15,29]. After rapid growth during the first two years of life, it is normal for a child’s growth to slow and for appetite to diminish accordingly [30]. Moreover, some toddlers begin asserting independence by self-feeding and becoming choosy about food. Such changes in eating habits may confuse parents and lead them to worry about their child as a picky eater.

A notable subset of children, however, have strong evidence of growth parameters and eating patterns indicating they are at risk for malnutrition [13,14,17,31]. Such malnutrition is concerning because the consequences of poor nutritional status with continuing growth faltering can be severe. Studies in populations of children in eight low- and middle-income countries [32], Southeast Asia [33], Egypt [34], Cambodia [35], and Tanzania [36] have shown links between malnutrition and growth faltering, which extend to impairment of cognition, communication, and motor development. Victora et al. reported lasting human costs related to the links between poor early nutrition and impaired brain development—decreased cognitive ability, less success in schooling, and lower adult productivity [37]. Malnutrition in children—protein calorie and micro-nutrient deficiencies—is a common underlying reason for increased susceptibility to respiratory and gastrointestinal infections [38] and increased risk of mortality [20]. Vulnerability to infectious pathogens is associated with malnutrition-related deficiencies in function of both innate and adaptive immune systems [18,39]. Studies also found that hospitalized pediatric patients have greater complication rates and higher costs when they are malnourished [40]. Further, a recent study examined people who had been picky eaters in childhood with mildly or transiently impaired growth; as young adults, they had lower intake of fruit, vegetables, and whole grains and more frequent intake of snack foods, sugar-sweetened beverages, and high-fat fast-foods, thus setting themselves up for poor health outcomes later in life [41]. Taken together, these studies provide a rationale to identify and intervene for inadequate nutrient intake, picky eating behaviors, and growth attenuation in young children. Insufficient or inadequate nutrition is the most common cause of growth attenuation in children. When food is replenished, spontaneous catch-up growth usually occurs, which brings the child back to his or her original growth trajectory [42]. For children who are underweight or short-for-age and poor eaters, intervention with oral nutrition supplements has been shown previously to improve outcomes. In a foundational study by Alarcon et al. [43], children who received nutritional supplements and whose parents underwent dietary counseling over a 90-day interval had significantly greater increases in weight and height than did those who received dietary counseling only. A recent study in India by Ghosh et al. showed that 90-day ONS + DC was effective for improving weight and reducing the incidence of respiratory tract infections in nutritionally at-risk, picky eating children with acute respiratory tract infection episodes [44]. Other studies of ONS for children with malnutrition likewise reported growth, health, and cost benefits [45,46,47]).

### 4.2. Strengths and Limitations of This Study

The design of this study in Indian children was strong because it was randomized, controlled, and blinded for ONS products used. Furthermore, the study design was broader than prior studies, as it was a three-arm design: dietary counseling plus one of two different ONS supplements versus dietary counseling alone. A high rate of compliance, completeness of dietary recall data, and a low number of subjects lost in follow-up are also strengths of this study. 

Results showed that a milk-based ONS and a lactose-free ONS were each more effective for promoting catch-up growth than was dietary counseling alone. As a limitation, the 3-month study interval was sufficient to observe catch-up growth in weight; however, the intervention interval should be longer, e.g., 6 months or more, to promote and observe catch-up growth in height.

Findings for this population of children in India may not be fully generalizable to all countries around the world because of the prominence of malnutrition as undernutrition in India. Nevertheless, shortfalls in growth of all undernourished young children have serious consequences and remain a concern worldwide.

## 5. Conclusions

Results of this randomized, controlled study showed that daily oral nutritional supplements can be used as an effective way to promote catch-up growth in young children who were at risk for malnutrition, including those who also have picky eating behaviors. In our study of catch-up growth, we examined the impact of two different pediatric ONS formulas—one milk-based and the other lactose-free—over a 90-day interval. Parents/guardians of children drinking ONS also underwent multiple sessions of dietary counseling; parents/guardians of a control group of children received dietary counseling only. The improved growth was similar for the two ONS formula groups, suggesting that both were similarly effective for promoting growth. Taken together, our findings confirm and extend prior studies in which ONS, along with dietary counseling, was more effective than dietary counseling alone to promote catch-up growth. We therefore see room for improvement worldwide in adopting and following health policies that recommend routine nutritional screening for all children; we also encourage enhanced training of professionals for identification and treatment of malnutrition [21,48].

## Figures and Tables

**Table 1:** Baseline characteristics for study population and treatment groups.

Characteristic	Total N = 321	ONS1 + DC N = 107	ONS2 + DC N = 107	DC Only N = 107	*p*-Value		
					ONS1 + DC vs. DC Only	ONS2 + DC vs. DC Only	ONS1 + DC vs. ONS2 + DC
Age (years) #	2.93 (2.47, 3.35)	2.94 (2.46, 3.38)	3.01 (2.47, 3.35)	2.92 (2.47, 3.37)	1.00	1.00	1.00
Male, n (%)	193 (60.1)	70 (65.4)	63 (58.9)	60 (56.1)	0.50	0.68	0.65
Female, n (%)	128 (39.9)	37 (34.6)	44 (41.1)	47 (43.9)			
Weight (kg) *	11.11 (1.55)	11.18 (1.51))	11.12 (1.58)	11.04 (1.57)	1.00	1.00	1.00
Height (cm) *	88.78 (7.09)	88.89 (6.97)	89.08 (7.19)	88.38 (7.15)	1.00	0.93	1.00
BMI (kg/m^2^) *	14.06 (0.58)	14.11(0.48)	13.98 (0.57)	14.10 (0.67)	0.98	0.05	0.09
MUAC (cm) *	14.08 (1.41)	14.11 (1.47)	14.00 (1.39)	14.11 (1.37)	0.84	0.78	0.84
**Z-scores**							
Weight-for-age *	−1.94 (0.98)	−1.92 (0.94)	−1.94 (1.01)	−1.97 (1.01)	1.00	1.00	1.00
Weight-for-height *	−1.46 (0.34)	−1.43 (0.28)	−1.52 (0.31)	−1.44 (0.42)	0.94	0.08	0.04
BMI-for-age *	−1.27 (0.45)	−1.24 (0.38)	−1.34 (0.45)	−1.24 (0.51)	0.99	0.05	0.06
Height-for-age *	−1.66 (1.65)	−1.67 (1.57)	−1.58 (1.71)	−1.74 (1.67)	1.00	0.88	1.00
MUAC-for-age *	−1.37 (1.26)	−1.36 (1.34)	−1.43 (1.25)	−1.33 (1.20)	1.00	0.90	1.00
**Percentiles**							
Weight-for-age #	2.60 (0.60, 9.30)	2.80 (0.50, 9.00)	2.40 (0.60, 8.60)	2.30 (0.50, 10.60)	1.00	1.00	1.00
Weight-for-height #	7.00 (4.40, 10.80)	7.20 (4.80, 11.80)	6.70 (3.90, 9.80)	7.40 (4.50, 11.30)	0.51	0.40	0.22
BMI-for-age #	9.60 (5.60, 15.00)	10.20 (5.90, 15.70)	8.20 (4.90, 14.30)	9.90 (5.90, 16.80)	0.86	0.30	0.27
Height-for-age #	4.90 (0.40, 29.10)	5.20 (0.30, 23.40)	5.60 (0.50, 29.20)	4.10 (0.30, 32.20)	1.00	1.00	1.00
MUAC-for-age #	9.00 (3.50, 26.60)	9.90 (4.50, 25.70)	8.40 (2.90, 28.70)	8.30 (2.70, 26.80)	1.00	1.00	1.00

* *p*-values are from analysis of covariance after adjusting for age, site, gender, and treatment with gender interaction. # *p*-values are from Wilcoxon rank sum test. Gender was analyzed using Cochran–Mantel–Haenszel test. All values are presented as Mean (SD) unless otherwise specified. For age and growth variables in percentiles, medians (Q1, Q3) are presented.

## Data Availability

Ethical restrictions imposed by the IRB prevents public sharing of the data for this study in children. The data used in this publication is owned by Abbott Nutrition. Data access request will be evaluated by Abbott Nutrition in consideration of IRB requirements. Interested researchers will need to sign a research collaboration agreement with Abbott. Requests can be sent to deepti.khanna@abbott.com.

## Supplementary Materials

The following are available online at https://www.mdpi.com/article/10.3390/nu13103590/s1, Table S1: Composition of Oral Nutritional Supplements ONS1 and 2, Table S2: Changes in weight (kg), height (cm), BMI (kg/m^2^) and MUAC (cm) across all time points, Table S3: Changes in weight-for-age, height-for-age, BMI-for-age, MUAC-for-age, and weight-for-height percentiles across all time points, Table S4: Changes in weight-for-age, height-for-age, BMI-for-age, MUAC-for-age, and weight-for-height Z-scores across all time points.
