# Supplementary material for: Oral Nutritional Supplementation Improves Growth in Children at Malnutrition Risk and with Picky Eating Behaviors
Source: Nutrients. 2021 Oct 14;13(10):3590. doi: 10.3390/nu13103590 (PMC8538528; doi:10.3390/nu13103590)
Supplement: Supplementary file 1 [file nutrients-13-03590-s001.zip › Table 1S.pdf]

Table 1S. Composition of Oral Nutritional Supplements (ONS) 1 and 2.

Abbreviations: CFU, colony-forming units; NE, RE, TE, niacin-, retinol-, tocopherol-equivalents

	Units	ONS1, Content per		ONS2, Content per	
		**100g**	**Serving**	**100g**	**Serving**
Energy	Kcal	462	210.2	468	212.9
Protein	g	14.1	6.4	14.1	6.4
Fat	g	17	7.7	23.4	10.6
Linoleic Acid	g	3.5	1.6	3.6	1.6
Linolenic Acid	g	0.38	0.2	0.4	0.2
Carbohydrate	g	62.7	28.5	50.21	22.8
Fructo-oligosaccharides	g	1.58	0.7	1.58	0.7
Taurine	mg	28	12.7	28	12.7
Carnitine	mg	6.7	3.0	6.7	3.0
Inositol	mg	32	14.6	32	14.6
Lactobacillus acidophilus	CFU	NA	NA	$1x10^8$	$45.5x10^6$
Bifidobacterium spp.	CFU	NA	NA	$1x10^8$	$45.5x10^6$
Vitamin A	mcg RE	305	138.8	305	138.8
Vitamin D2	mcg	3.15	1.4	3.15	1.4
Vitamin E	mg α-TE	11	5.0	11	5.0
Vitamin K	mcg	17.5	8.0	17.5	8.0
Vitamin C	mg	44	20.0	44	20.0
Folic Acid	mcg	100	45.5	100	45.5
Vitamin B1	mg	0.9	0.4	0.9	0.4
Vitamin B2	mg	1	0.5	1	0.5
Vitamin B6	mg	1	0.5	1	0.5
Vitamin B12	mcg	1.5	0.7	1.5	0.7
Niacin	mg-NE	7	3.2	7	3.2
Pantothenic Acid	mg	3.1	1.4	3.1	1.4
Biotin	mcg	16	7.3	16	7.3
Choline	mg	118	53.7	118	53.7
Sodium	mg	181	82.4	181	82.4

Potassium	mg	512	233.0	512	233.0
Chloride	mg	394	179.3	394	179.3
Calcium	mg	386	175.6	386	175.6
Phosphorus	mg	240	109.2	240	109.2
Magnesium	mg	78	35.5	78	35.5
Iron	mg	5.5	2.5	5.5	2.5
Zinc	mg	3.5	1.6	3.5	1.6
Manganese	mg	0.98	0.4	0.98	0.4
Copper	mcg	400	182.0	400	182.0
Iodine	mcg	38	17.3	38	17.3
Selenium	mcg	12.3	5.6	12.3	5.6
Chromium	mcg	12	5.5	12	5.5
Molybdenum	mcg	19.7	9.0	19.7	9.0
