# Supplementary material for: Oral Nutritional Supplementation Improves Growth in Children at Malnutrition Risk and with Picky Eating Behaviors
Source: Nutrients. 2021 Oct 14;13(10):3590. doi: 10.3390/nu13103590 (PMC8538528; doi:10.3390/nu13103590)
Supplement: Supplementary file 1 [file nutrients-13-03590-s001.zip › Table 2S.pdf]

Table 2S. Changes in weight (kg), height (cm), BMI (kg/m^2) and MUAC (cm) across all time points. Green shading represents p values that are statistically significant differences.

Anthropometric measures Median (Q1, Q3)	Change across time points from	ONS1 + DC	ONS2 + DC	DC only	P value		
					ONS1 + DC vs ONS2 + DC	ONS1 + DC vs DC only	ONS2 + DC vs DC only
**Weight**	Day 1 to 7	0.13 (0.00,0.30)	0.10 (0.00,0.23)	0.10 (0.00,0.20)	0.2536	0.0222	0.3277
	Day 1 to 30	0.40 (0.20,0.70)	0.40 (0.17,0.70)	0.30 (0.10,0.50)	0.5164	0.0015	0.0140
	Day 1 to 60	0.70 (0.40,1.10)	0.60 (0.30,1.10)	0.50 (0.30,0.70)	0.3187	0.0043	0.1377
	Day 1 to 90	0.88 (0.58,1.35)	0.80 (0.60,1.30)	0.62 (0.45,0.90)	0.9517	0.0012	0.0012
**Height**	Day 1 to 30	0.20 (0.00,0.80)	0.15 (0.00,0.50)	0.10 (0.00,0.50)	1.0000	0.9184	1.0000
	Day 1 to 60	0.50 (0.10,1.55)	0.50 (0.10,1.50)	0.30 (0.05,1.35)	1.0000	1.0000	1.0000
	Day 1 to 90	0.90 (0.20,2.18)	0.70 (0.20,2.30)	0.60 (0.15,1.95)	0.7910	0.5257	0.7910
**BMI**	Day 1 to 30	0.39 (0.16,0.73)	0.35 (0.15,0.77)	0.27 (0.12,0.47)	0.8020	0.0172	0.0358
	Day 1 to 60	0.62 (0.36,1.00)	0.54 (0.28,1.00)	0.43 (0.23,0.74)	0.5625	0.0075	0.0746
	Day 1 to 90	0.73 (0.45,1.35)	0.74 (0.50,1.33)	0.58 (0.32,0.89)	0.5829	0.0185	0.0064
**MUAC**	Day 1 to 30	0.10 (0.00,0.30)	0.10 (0.00,0.20)	0.05 (0.00,0.15)	0.1685	0.0949	0.6532
	Day 1 to 60	0.28 (0.10,0.60)	0.20 (0.05,0.40)	0.10 (0.00,0.30)	0.3410	0.0230	0.3410
	Day 1 to 90	0.34 (0.10,0.78)	0.30 (0.10,0.70)	0.20 (0.05,0.50)	0.4195	0.0418	0.2311
