# Supplementary material for: Oral Nutritional Supplementation Improves Growth in Children at Malnutrition Risk and with Picky Eating Behaviors
Source: Nutrients. 2021 Oct 14;13(10):3590. doi: 10.3390/nu13103590 (PMC8538528; doi:10.3390/nu13103590)
Supplement: Supplementary file 1 [file nutrients-13-03590-s001.zip › Table 3S.pdf]

Table 3S. Changes in weight-for-age, height-for-age, BMI-for-age, MUAC-for-age, and weight-for-height percentiles across all time points. Green shaded boxes highlight statistically significant differences.

Anthropometric indices Median (Q1, Q3)	Change across time points from	ONS1 + DC	ONS2 + DC	DC only	P value		
					ONS1 + DC vs ONS2 + DC	ONS1 + DC vs DC only	ONS2 + DC vs DC only
Weight-for-age	Day 1 to 7	0.30 (0.00,1.80)	0.20 (-0.10,1.60)	0.20 (0.00,0.90	0.6878	0.3179	0.6878
	Day 1 to 30	0.80 (0.10,3.70)	0.60 (0.00,3.30)	0.40 (0.00,1.60)	0.5071	0.0489	0.1540
	Day 1 to 60	1.30 (0.30,5.60)	0.90 (0.00,5.80)	0.60 (0.00,3.00)	0.3222	0.0139	0.3222
	Day 1 to 90	1.65 (0.30,8.10)	2.40 (0.10,7.80)	0.70 (0.00,2.80)	0.9631	0.0054	0.0054
Height-for-age	Day 1 to 30	-0.15 (-2.00,0.10)	-0.30 (-2.50,0.00)	-0.20 (-3.00,0.00)	0.4728	0.4728	0.9800
	Day 1 to 60	-0.10 (-3.80,0.30)	-0.20 (-3.40,0.20)	-0.20 (-3.00,0.10)	1.0000	1.0000	1.0000
	Day 1 to 90	-0.10 (-3.75,0.50)	-0.30 (-6.10,0.20)	-0.20 (-5.10,0.00)	0.4323	0.4323	0.8382
BMI-for-age	Day 1 to 30	9.55 (3.00,16.40)	7.10 (2.50,16.40)	4.70 (2.10,10.10)	0.5369	0.0048	0.0523
	Day 1 to 60	14.85 (6.80,24.90)	12.10 (4.50,25.90)	8.90 (4.80,18.00)	0.5278	0.0098	0.1182
	Day 1 to 90	18.35 (9.00,36.85)	18.75 (7.60,37.65)	13.50 (6.50,23.50)	0.9206	0.0214	0.0203
MUAC-for-age	Day 1 to 30	0.60 (-0.40,4.40)	0.10 (-0.50,1.60)	0.00 (-0.50,1.60)	0.3512	0.1368	0.4851
	Day 1 to 60	1.60 (0.00,10.00)	1.10 (0.00,8.80)	0.20 (-0.80,3.40)	0.4940	0.0342	0.1445
	Day 1 to 90	2.65 (0.00,13.45)	2.95 (0.00,11.60)	0.50 (-0.80,5.40)	0.5356	0.0241	0.1071
Weight-for-height	Day 1 to 30	7.35 (2.20,12.70)	5.30 (2.00,13.50)	3.70 (1.50,7.10)	0.5157	0.0036	0.0493
	Day 1 to 60	0.58 (0.36,0.95)	0.53 (0.32,0.93)	0.42 (0.25,0.69)	0.7850	0.0063	0.0205
	Day 1 to 90	14.85 (6.60,28.20)	15.15 (6.65,28.55)	9.50 (5.30,17.60)	0.9348	0.0086	0.0086
