# Supplementary material for: Oral Nutritional Supplementation Improves Growth in Children at Malnutrition Risk and with Picky Eating Behaviors
Source: Nutrients. 2021 Oct 14;13(10):3590. doi: 10.3390/nu13103590 (PMC8538528; doi:10.3390/nu13103590)
Supplement: Supplementary file 1 [file nutrients-13-03590-s001.zip › Table 4S.pdf]

Table 4S. Changes in weight-for-age, height-for-age, BMI-for-age, MUAC-for-age, and weight-for-height Z-scores across all time points. Green-shaded boxes highlight statistically significant differences.

Anthropometric indices Median (Q1, Q3)	Change across time points from	ONS1 + DC	ONS2 + DC	DC only	P value		
					ONS1 + DC vs ONS2 + DC	ONS1 + DC vs DC only	ONS2 + DC vs DC only
Weight-for-age	Day 1 to 7	0.08 (-0.02,0.19)	0.05 (-0.02,0.14)	0.04 (-0.02,0.10)	0.2505	0.0165	0.3234
	Day 1 to 30	0.21 (0.05,0.42)	0.17 (0.02,0.42)	0.10 (-0.01,0.23)	0.5141	0.0018	0.0128
	Day 1 to 60	0.30 (0.10,0.56)	0.24 (0.06,0.56)	0.19 (0.03,0.32)	0.3859	0.0056	0.1324
	Day 1 to 90	0.36 (0.11, 0.70)	0.31 (0.17, 0.61)	0.21 (0.05, 0.37)	0.8905	0.0015	0.0020
Height-for-age	Day 1 to 30	-0.10 (-0.15,0.05)	-0.10 (-0.15,0.00)	-0.11 (-0.16, -0.03)	1.0000	1.0000	1.0000
	Day 1 to 60	-0.19 (-0.29,0.11)	-0.18 (-0.28,0.07)	-0.18 (-0.28,0.03)	1.0000	1.0000	1.0000
	Day 1 to 90	-0.20 (-0.39,0.12)	-0.27 (-0.40,0.07)	-0.28 (-0.38,0.02)	1.0000	1.0000	1.0000
BMI-for-age	Day 1 to 30	0.37 (0.17,0.68)	0.34 (0.17,0.71)	0.27 (0.13,0.46)	0.8901	0.0150	0.0334
	Day 1 to 60	0.57 (0.38,0.93)	0.52 (0.29,0.91)	0.42 (0.25,0.69)	0.5338	0.0067	0.0750
	Day 1 to 90	:0.71 (0.48,1.15)	0.75 (0.53,1.19)	0.60 (0.34,0.81)	0.4937	0.0196	0.0044
MUAC-for-age	Day 1 to 30	0.06 (-0.03,0.21)	0.03 (-0.03,0.14)	0.00 (-0.04,0.13)	0.1572	0.0929	0.6458
	Day 1 to 60	0.17 (0.02,0.43)	0.10 (0.00,0.30)	0.04 (-0.06,0.23)	0.2722	0.0192	0.2722
	Day 1 to 90	0.24 (0.03,0.56)	0.17 (-0.01,0.45)	0.11 (-0.04,0.30)	0.4744	0.0391	0.1714
Weight-for-height	Day 1 to 30	0.34 (0.15,0.65)	0.32 (0.14,0.69)	0.24 (0.11,0.41)	0.8654	0.0156	0.0299
	Day 1 to 60	0.54 (0.34,0.92)	0.48 (0.26,0.89)	0.40 (0.20,0.63)	0.5270	0.0056	0.0707
	Day 1 to 90	0.66 (0.43,1.10)	0.70 (0.46,1.10)	0.53 (0.28,0.78)	0.5992	0.0138	0.0046
